# Impact of room acoustics and visual cues on speech perception and talker localization by children with mild bilateral or unilateral hearing loss

**DOI:** 10.3389/fped.2023.1252452

**Published:** 2023-11-22

**Authors:** Dawna Lewis, Sarah Al-Salim, Tessa McDermott, Andrew Dergan, Ryan W. McCreery

**Affiliations:** ^1^ Listening and Learning Laboratory, Boys Town National Research Hospital, Omaha, NE, United States; ^2^ Auditory Perception and Cognition Laboratory, Boys Town National Research Hospital, Omaha, NE, United States; ^3^ Clinical Measurement Program, Boys Town National Research Hospital, Omaha, NE, United States

**Keywords:** unilateral hearing loss, mild bilateral hearing loss, speech perception, audiovisual, room acoustics

## Abstract

**Introduction:**

This study evaluated the ability of children (8–12 years) with mild bilateral or unilateral hearing loss (MBHL/UHL) listening unaided, or normal hearing (NH) to locate and understand talkers in varying auditory/visual acoustic environments. Potential differences across hearing status were examined.

**Methods:**

Participants heard sentences presented by female talkers from five surrounding locations in varying acoustic environments. A localization-only task included two conditions (auditory only, visually guided auditory) in three acoustic environments (favorable, typical, poor). Participants were asked to locate each talker. A speech perception task included four conditions [auditory-only, visually guided auditory, audiovisual, auditory-only from 0° azimuth (baseline)] in a single acoustic environment. Participants were asked to locate talkers, then repeat what was said.

**Results:**

In the localization-only task, participants were better able to locate talkers and looking times were shorter with visual guidance to talker location. Correct looking was poorest and looking times longest in the poor acoustic environment. There were no significant effects of hearing status/age. In the speech perception task, performance was highest in the audiovisual condition and was better in the visually guided and auditory-only conditions than in the baseline condition. Although audiovisual performance was best overall, children with MBHL or UHL performed more poorly than peers with NH. Better-ear pure-tone averages for children with MBHL had a greater effect on keyword understanding than did poorer-ear pure-tone averages for children with UHL.

**Conclusion:**

Although children could locate talkers more easily and quickly with visual information, finding locations alone did not improve speech perception. Best speech perception occurred in the audiovisual condition; however, poorer performance by children with MBHL or UHL suggested that being able to see talkers did not overcome reduced auditory access. Children with UHL exhibited better speech perception than children with MBHL, supporting benefits of NH in at least one ear.

## Introduction

Children with mild bilateral hearing loss (MBHL) or unilateral hearing loss (UHL) make up at least 5% of school-age children in the United States (1, 2), representing approximately 2.5 million children from pre-kindergarten through 12th grade (3). Children with MBHL or UHL are typically educated in mainstream classrooms alongside peers with normal hearing (NH), in acoustic environments that often do not meet recommended standards for children with hearing loss (4–8). Spratford et al. (8) tested noise and reverberation in 164 general education classrooms. They reported that 87.3% of the classrooms had unoccupied noise levels above the recommended level of 35 dBA. Reverberation times were above the 0.3 s recommended for classrooms educating children who are deaf/hard of hearing or have other communication issues in 62.2% of classrooms.

In classrooms where children are learning, acoustic environments change often, with a variety of talkers and noise sources around the classroom that fluctuate in level. To hear and understand talkers in these environments, children will need to identify and separate the ones they want to listen to from other voices and sounds in the environment. They also may need to quickly shift their attention among multiple sound sources. Depending on the task, children with MBHL or UHL may perform more poorly than children with NH when attempting to understand speech in noise and reverberation (1, 9–15). However, much of the research examining speech understanding in children with MBHL or UHL has not taken real-world listening conditions into account. Not doing so could result in overestimations of how these children will perform in real listening conditions, which could, in turn, impact the provision of educational services that would support listening and learning for children with MBHL and UHL in general education classrooms (16, 17).

When acoustics make it difficult to hear speech, seeing a talker's face can improve children's speech understanding—a skill that improves with age (18–21). As a result of reduced auditory access, children with MBHL or UHL may depend on these visual cues more than children with NH. Recent work by Lalonde and McCreery (18) revealed that school-age children who were hard of hearing exhibited greater audiovisual benefit for sentence recognition in noise than children with NH. Being able to quickly locate talkers to see their faces may strengthen speech understanding in classrooms with poor acoustics and multiple talkers. However, a challenge exists for children with MBHL or UHL since the effort to locate talkers in the presence of reduced auditory access may use cognitive effort that might otherwise be used for speech understanding and learning.

Although children with MBHL or UHL may demonstrate similar difficulties in speech understanding in noise and reverberation, speech and language development, and academic performance (1, 12, 13, 15, 17, 22–31), the underlying mechanisms for these difficulties are likely to be different. For children with UHL, access to binaural cues can be reduced or absent depending on the degree of hearing loss in the poorer ear. Binaural cues are used for locating and separating auditory signals, benefiting speech understanding in background noise (32). Reduced access to these cues may negatively impact speech understanding in children with UHL, particularly for talkers from the direction of their poorer hearing ear. Children with MBHL have access to binaural cues, particularly when hearing levels are symmetrical across ears. However, they experience reduced access to signals from both ears when compared to listeners with NH or UHL. Poor access to speech signals may hinder how well children with MBHL or UHL understand talkers.

To address the conditions children will experience in the real world, numerous studies since the early 2000s have assessed speech understanding in children with NH and children with MBHL or UHL using complex listening tasks and acoustic conditions (12, 33–37). For example, Griffin et al. (33) reported that even when presentation levels were individualized based on sentence-recognition performance, children with UHL performed more poorly than children with NH on a comprehension task. Lewis et al. (12) used audiovisual tasks of speech understanding designed to simulate plausible listening conditions in a classroom to examine the impact of MBHL and UHL on sentence recognition and comprehension. Children with NH and children with MBHL or UHL were tested using a traditional single talker auditory-only sentence recognition task and an audiovisual comprehension task presented by multiple talkers, both presented from multiple locations. Overall, sentence recognition scores were high across all groups, suggesting little impact of hearing status. For the comprehension task, children with MBHL or UHL performed more poorly than those with NH but there were no differences in performance for the two hearing-loss groups. These findings suggested that complex listening tasks in realistic acoustic environments can negatively affect speech understanding in children with MBHL or UHL to a greater extent than children with NH.

The current study was designed to further examine the ability of children with MBHL or UHL to locate and understand talkers under a range of conditions, with a goal of differentiating performance across hearing status groups using tasks that were less complex than our previous comprehension tasks but more complex than simple sentence recognition tasks.

Visual cues directing a listener to the location of a sound can improve identification of that sound for adults with NH or hearing loss (38, 39). Visually guiding children with MBHL or UHL to the talker's location has the potential to reduce effort required to locate that talker as the acoustic environment varies but has not been examined to date. However, locating a talker, even in adverse acoustics, may not require as much effort as locating that talker and understanding what they are saying. Two tasks were used address children's ability to locate talkers and understand them in complex listening conditions, In a localization-only task, children with NH and children with MBHL or UHL were asked to locate talkers under auditory-only and visually guided auditory conditions in three different acoustic environments that children might experience in classrooms. In a speech perception task, children with NH and children with MBHL or UHL were asked to locate multiple talkers and repeat back what each talker said under varying auditory and auditory-visual conditions in a single acoustic environment.

This experiment addressed the following research questions.

1. *Does acoustic environment impact the ability of children with MBHL, UHL, or NH to locate talkers in auditory-only* vs. *visually guided conditions and how does performance compare across groups?*
2. *Does acoustic environment impact looking time of children with MBHL, UHL, or NH who correctly locate talkers in auditory-only and visually guided conditions and how does performance compare across groups?*
3. *Do auditory and visual accessibility impact speech perception for children with MBHL, UHL, or NH and how does performance compare across groups?*
4. *For children with MBHL or UHL, do audiological (audibility in better (MBHL) or poorer (UHL) ear) and cognitive (vocabulary, working memory) factors help to explain individual differences in speech perception?*

## Methods

### Test environment and stimuli

**Figure 1:**
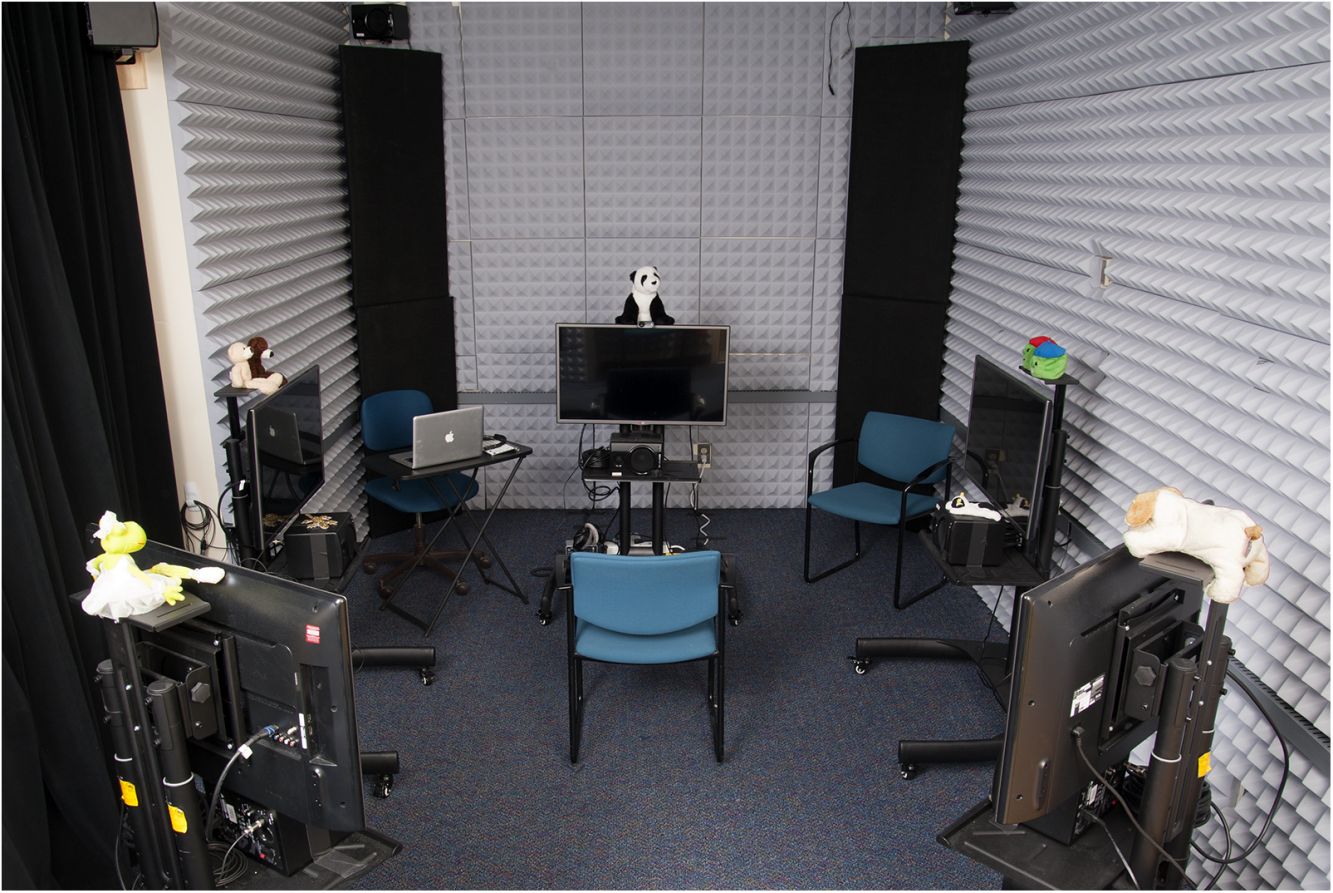
Experimental set-up.

A simulated acoustic environment was created following the procedures described in Valente, et al. (37). The simulated room was acoustically treated with acoustic wall and ceiling tiles, carpeting, and a velour curtain. The unaltered acoustic environment in the test space had a 37.4 dBA LEQ background noise level and a 0.18 s reverberation time (T30 mid). As previously described in Salanger et al. (40), participants were seated in the center of the test space surrounded by stands with five 32-inch high-definition televisions (HDTVs; Samsung Syncmaster 2,433) and loudspeakers [M-Audio Studiophile AV (40)] that were arranged around the participant's location at 0^o^, 90^o^, 121^o^, −121^o^, and −90^o^ (Figure 1). Virtual microphone control [ViMiC (41)], generated the simulated environment. Speech-shaped noise was radiated incoherently through the five loudspeakers. The direct sound and first-order reflections were processed through ViMiC and combined with late reverberation and speech shaped noise to create the simulated acoustic space. The audio signals were positioned in a virtual room model to simulate appropriate source distance, reflections, and reverberation.

A custom-built wireless attitude and heading reference system (AHRS) tracked participants' head movements. Head movements were processed in real time using a microcontroller, to provide attitude and heading solutions as Euler angles over Bluetooth.

Stimuli consisted of 96 five-to-six-word low-predictability sentences, video-recorded by four adult female talkers of American English. The sentences were syntactically correct but semantically incorrect with four keywords each (e.g., “The collar charged the silly cement”, “The magic ceilings guess far”; keywords underlined) that were chosen to be within the lexicon of children in the first grade (42).

### Clinical assessments

Clinical assessments were administered by audiologists and speech-language pathologists who had experience working with children with hearing loss. Audiometric thresholds were measured by an audiologist in a sound-treated, double-walled booth. The Automated Working Memory Assessment [AWMA (43)] Odd One Out subtest was used to measure visuo-spatial working memory. In this task, the child must first indicate the “odd one out” or different shape from a set of three shapes and then recall the position of the different shape on an empty grid. The number of grids in each sequence increases when the child attains four correct answers in a set of six sequences. The Peabody Picture Vocabulary Test–4 [PPVT-4 (44)] was used to assess receptive vocabulary. In the PPVT-4, the child selects a picture that matches a target word from a set of four choices. Visual acuity of all participants was screened using a Sloan letters chart (45). Participants with prescription glasses or contacts were required to wear them during the screening. To pass the screening, the participant must have had a visual acuity screening threshold of 20/32 or better in both eyes.

### Participant*s*

Forty children with NH [21 male (52.5%)], 21 children with MBHL [10 male (47.6%)], and 17 children with UHL [12 male (70.6%)] participated. The number of participants was motivated by a power analysis for main effects by group. Children were included if their age was within three months of the target age range of 8 to 12 years. The mean age for the children with NH was 10.5 years (range: 8.1–13.0). For the children with MBHL, the mean age was 10.3 years (range: 8.1–12.8) and for the children with UHL it was 10.0 years (range: (7.9–13.3). Twenty of the children with NH participated in the localization-only task and 20 participated in the speech recognition task. Although not required, all except two children with MBHL or UHL participated in both tasks; one child with MBHL participated only in the localization-only task and one child with UHL participated only in the speech recognition task.

For the current study, children were considered to have NH if their air-conducted thresholds were 15 dB HL or better at all octave frequencies 250–8,000 Hz in both ears. MBHL was defined as a 4-frequency better-ear pure-tone average (BEPTA;.5, 1, 2, 4 kHz) threshold >20 and ≤45 dB HL or thresholds >25 dB HL at one or more frequencies above 2 kHz in both ears.

For 20 of the children with MBHL, the mean BEPTA was 33.7 dB HL (SD = 7.14). One participant with MBHL had a high-frequency hearing loss, with a BEPTA for the frequencies with hearing loss (6–8 kHz) of 67.5 dB HL. Children with MBHL presented with sensorineural hearing loss in both ears (*n* = 16), conductive hearing loss in both ears (*n* = 2), mixed hearing loss in both ears (*n* = 2), sensorineural hearing loss in one ear and mixed hearing loss in the other (*n* = 1), or undetermined (*n* = 1).

Unilateral hearing loss was defined as a 4-frequency pure-tone average threshold >20 dB HL in the poorer ear (PEPTA) and <20 dB HL in the better ear, or thresholds >25 dB HL at one or more frequencies above 2 kHz and ≤15 dB HL at frequencies below 2 kHz in the poorer ear. Eight children had UHL in the right ear and nine had UHL in the left ear. For 15 of the children with UHL, the PEPTA was 58.7 dB HL (SD = 29.9). One of those participants did not have thresholds in the poorer ear that were within the limits of the audiometer. For analysis purposes, that participant's PEPTA was included as 125 dB HL. Two participants with UHL presented with high-frequency hearing loss. For one of those participants, the PEPTA for the frequencies with hearing loss was 40 dB HL (4 kHz, left), and for the other it was 40 dB HL (3, 6, 8 kHz, right). In the poorer ear, children with UHL presented with sensorineural hearing loss (*n* = 9), conductive hearing loss (*n* = 4), mixed hearing loss (*n* = 2), or undetermined (*n* = 2).

Audiological, vocabulary, and working memory characteristics of participants with MBHL and UHL are summarized in Table 1. Age of onset of hearing loss and possible progression of hearing loss for children with MBHL or UHL were not available. Testing was completed without personal hearing aids.

**Table 1:** Audiological characteristics, vocabulary, and working memory for participants with MBHL and UHL.

	MBHL	UHL
Age of identification (months)	M = 36.3	M = 35.8
	Mdn = 36.0	Mdn = 24.0
	Range = 0–108	Range = 1–108
Better ear PTA (MBHL; dB HL)	M = 32.5 Mdn = 32.5	
	Range = 8.8–42.5	
Poorer ear PTA (UHL; dB HL)		M = 58.2
		Mdn = 46.3
		Range = 12.5–125
Fitted with at least 1 HA	17 [81%]	11 [64.7%]
Type of hearing aid	Bilateral BTEs (15)	BTE (10)
		CROS (1)
	Unilateral BTE (1)	
	Unilateral bone-anchored device (1)	
Age of initial HA fitting (months)	M = 48.3	M = 60.7
	Mdn = 51.5	Mdn = 60.0
	Range = 2–96	Range = 22–108
Language (PPVT)	M = 108.52	M = 112.13
	SD = 14.37	SD = 9.63
Working memory (Odd One Out; AWMA)	M = 110.40	M = 113.31
	SD = 19.68	SD = 13.94

NH, normal hearing; MBHL, mild bilateral hearing loss; UHL, unilateral hearing loss; M, mean; Mdn, median; SD, standard deviation; PTA, pure-tone average; HA, hearing aid; BTE, behind-the-ear; CROS, contralateral routing of signals; PPVT, peabody picture vocabulary test; AWMA, automated working memory test.

### Procedures

For the localization-only and speech perception tasks, sentences were presented randomly by the four talkers from each of the five locations around the listener, at 60 dBA. Conditions were randomized for each task and sentence order and talker within conditions were randomized within tasks.

Looking behavior was monitored using the AHRS to assess both speed and accuracy of localization. Pilot testing determined the minimum angle (in degrees relative to 0^o^ azimuth) at which head turn plus eye turn toward a loudspeaker and screen would allow participants to visualize each of the five screens. Minimum angles for the four non-zero-degree locations were determined to be ±30^o^ (for loudspeakers at ±90^o^ turning right/left), ±85^o^ (for loudspeakers at ±121^o^ turning right/left).

Participants could move their upper body to allow for more natural looking behaviors. Localization was recorded as angular data in the horizontal plane. Looking accuracy was coded as correct when the participant looked into the region for the loudspeaker/screen of the target talker but did not look past that region. If the participant did not look in the correct region or he/she moved beyond that region, accuracy was coded as incorrect. Looking time was analyzed only for those trials coded as correct for looking accuracy.

#### Localization-only task

Participants heard sentences presented in two conditions (auditory only, visually guided auditory) and three acoustic environments (favorable, typical, poor). In the auditory-only condition, no visual cues were available. In the visually guided condition, the TV screen located above a loudspeaker illuminated blue if the sentence was presented from that loudspeaker. Acoustic environments were chosen to represent a range of listening environments for classroom listening: *Favorable* (noise = 22 dB signal-to-noise ratio [SNR], reverberation time [T30 mid] = 0.5 s); *Typical* (noise = 6 dB SNR, T30 mid = 0.7 s); and *Poor* (noise = 0 dB SNR, T30 mid = 1.3 s). Listeners were instructed to look at the talker's location as quickly as possible after she began speaking. After locating each talker, participants were required to return to the 0^o^ azimuth position before the next sentence was presented.

#### Speech perception task

Participants listened to sentences presented under four randomized conditions: (1) auditory-only, (2) visually guided auditory (3) audiovisual, (4) baseline (single location auditory-only at 0^o^ azimuth). In the two auditory-only conditions, no visual cues were available. The visually guided condition was the same as in the localization-only task. For the audiovisual condition, recordings of the talkers were presented on the HDTVs using custom software developed in Max 6.

Reverberation for the speech perception task was T30 mid = 0.6 s and SNRs were 0 dB or 3 dB for children with NH or MBHL/UHL, respectively. The different SNRs for NH vs. MBHL/UHL participants were chosen to allow a range of speech-perception performance levels for all groups without ceiling or floor effects.

For the auditory only, visually guided auditory, and audiovisual conditions, sentences were presented randomly by one of four talkers from each of the five locations around the listener. For the baseline condition, each of the four talkers was presented randomly from the speaker at 0^o^ azimuth. For all conditions except baseline, listeners were asked to locate the talker as quickly as possible as each sentence was presented and then repeat the sentence. After locating each talker, participants were to return to face the 0^o^ azimuth position. For the baseline condition, they were asked to look forward throughout the condition. Responses were scored by number of keywords correct (keyword scoring) and by whether all keywords were correct (sentence scoring). A researcher scored the sentences as they were administered. Responses also were video recorded to allow the researcher to recheck scores.

### Statistical methods

Linear mixed effects models were conducted using R Statistical Software [R Core Team, v. 4.1.3 (46)] and the lme4 (47) and lmerTest (48) packages. Figures were created using the ggplot2 package [v.3.3.5 (49)]. Descriptive statistics for each group were calculated. Pearson correlations were calculated for children with MBHL or UHL for variables that were only collected for children with hearing loss including: age of hearing loss identification (in months), audiological (better-ear and poorer-ear PTA for mild bilateral and unilateral participants, respectively), language [PPVT-4 (44)], and working memory [Odd One Out subtest of the AWMA (43)]. All linear mixed effects models included a random intercept for each participant to account for correlations between repeated measures within the same participants. Effects are reported as raw coefficients to support interpretation of effects.

Separate models were used in each experiment to examine percent correct looking, looking time, and speech perception. In the localization-only task, the fixed effects were age (in years), acoustic condition (favorable, typical, and poor), audiovisual cues (auditory-only or visually guided), and hearing group (NH, UHL, and MBHL). The auditory-only, favorable condition was coded as the reference in the localization-only task model. In the speech perception task, the fixed effects were age (in years), condition (auditory-only, visually guided, audiovisual, and single location baseline), and hearing group (NH, UHL, and MBHL) with the single-location baseline coded as the reference condition. For both models, hearing group was coded in contrast to the children with MBHL. Model assumptions were confirmed by examining the normality of the distribution of model residuals. *Post-hoc* tests for significant main effects with multiple comparisons were interpreted with *p*-values adjusted using the False Discovery Rate procedure to control for Type I error rate with multiple comparisons (50).

## Results

*Does acoustic environment impact the ability of children with MBHL, UHL, or NH to locate talkers in auditory-only* vs. *visually guided conditions and how does performance compare across groups?*

**Figure 2:**
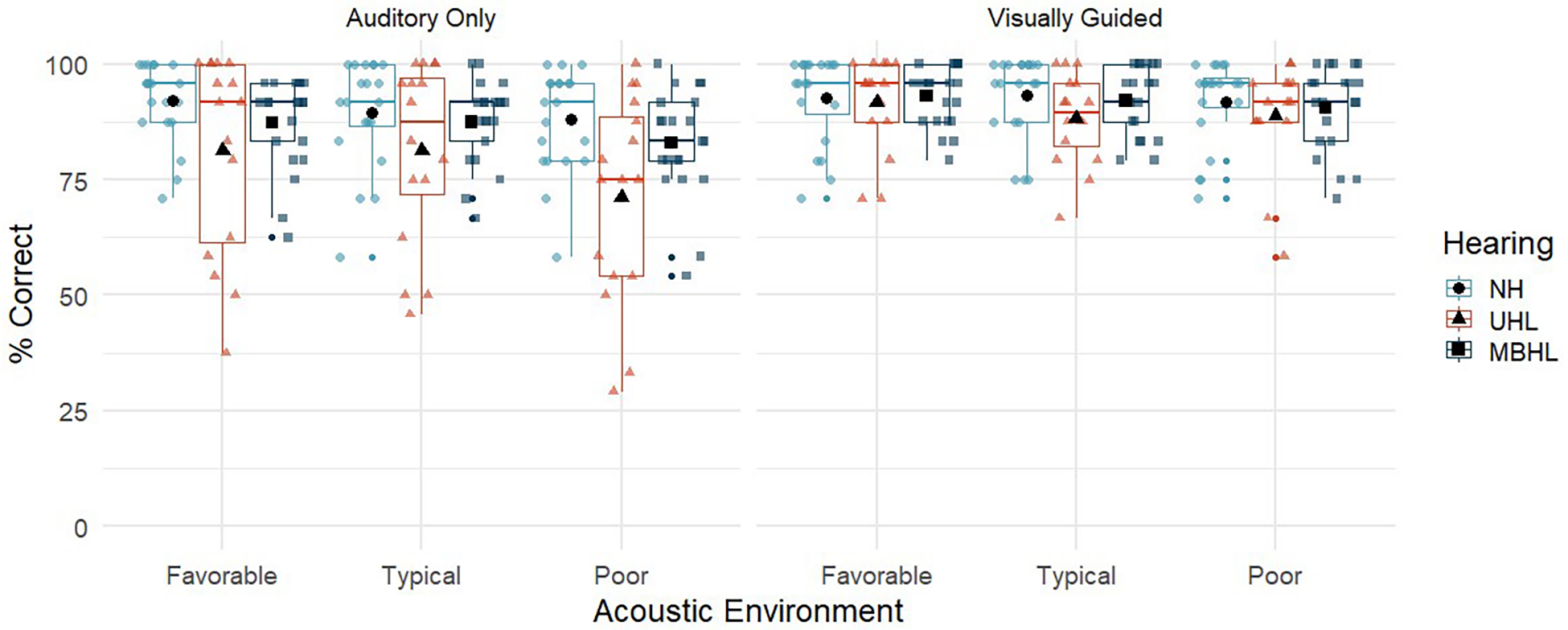
Percent correct looking for children with normal hearing (NH; light blue circles), children with unilateral hearing loss (UHL; red triangles) and children with mild bilateral hearing loss (MBHL, dark blue squares) for the three acoustic environments (favorable, typical, poor). Results are shown for the Auditory-Only (left panel) and Visually Guided (right panel) conditions. Boxes represent the interquartile range, and whiskers represent the 5th and 95th percentiles. For each box, lines represent the median. Colored symbols represent individual data points. Black filled symbols represent means.

The initial analyses addressed percent correct looking by acoustic environment and auditory/visual cues for children with NH, UHL, or MBHL (Figure 2 and Table 2). Table 3 shows the statistics for the linear mixed effects model for percent correct looking. The main effects of acoustic environment and auditory/visual cues on percent correct looking were significant, but none of the differences between hearing groups or higher-order interactions were statistically significant. *Post-hoc t*-tests showed that percent correct looking in the poor acoustic environment was poorer than in the typical (Coefficient = −5.9, *p* < 0.001) and favorable (Coefficient = −6.8, *p* < 0.001) environments, but the difference between typical and favorable environments was not significant (Coefficient = −0.88, *p* = 0.58). Visually guided conditions had higher percent correct looking than auditory-only conditions (Coefficient = 5.3, *p* < 0.001). The condition by hearing group interaction was not significant (Coefficient = 0.72, *p* = 0.58).

**Table 2:** Mean (standard deviation) for percent correct looking by group and listening condition.

Condition	NH	UHL	MBHL
Favorable (AO)	92.1 (8.7)	81.3 (21.5)	87.3 (9.8)
Favorable (VG)	92.7 (9.6)	91.7 (10.0)	93.1 (6.5)
Typical (AO)	89.4 (11.7)	81.3 (19.7)	87.5 (8.9)
Typical (VG)	93.3 (9.0)	88.3 (9.6)	92.1 (7.2)
Poor (AO)	87.9 (11.1)	71.1 (22.2)	82.9 (11.6)
Poor (VG)	91.9 (9.3)	88.9 (11.6)	90.5 (9.2)

AO, auditory only; VG, visually guided; NH, normal hearing, UHL, unilateral hearing loss; MBHL, mild bilateral hearing loss.

**Table 3:** Linear mixed effects model for group by condition.

Predictors	Percent correct looking		
	Estimates	CI	*p*
(Intercept)	79.41	62.55–96.27	**<0**.**001**
Poor vs. favorable	−5.92	−9.01 to −2.84	**<0**.**001**
Typical vs. favorable	−0.88	−3.96 to 2.21	0.576
AO vs. VG	5.26	2.17–8.34	**0**.**001**
NH vs. MBHL	2.22	−3.50 to 7.94	0.446
UHL vs. MBHL	−4.79	−10.92 to 1.34	0.125
Age (years)	0.83	−0.75 to 2.40	0.303
Condition/AV interaction	3.77	−0.60 to 8.15	0.091
Condition/hearing group interaction	0.72	−2.58 to 3.61	0.58
Random effects			
*σ* ^2^	70.10		
*τ*_00__ID_	74.82		
ICC	0.52		
N_ID_	57		
Observations	341		
Marginal R^2^/conditional R^2^	0.147/0.588		

AO, auditory only; VG, visually guided; NH, normal hearing, UHL, unilateral hearing loss; MBHL, mild bilateral hearing loss.

Estimates represent the coefficients for each variable in the model. For categorical predictors, the estimate represents the mean difference. For continuous predictors, the estimate represents the change in looking time for a one unit change in the predictor.

All *p*-values for significant effects are bolded.

*Does acoustic environment impact looking time of children with MBHL, UHL, or NH who correctly locate talkers in auditory-only and visually guided conditions and how does performance compare across groups?*

**Figure 3:**
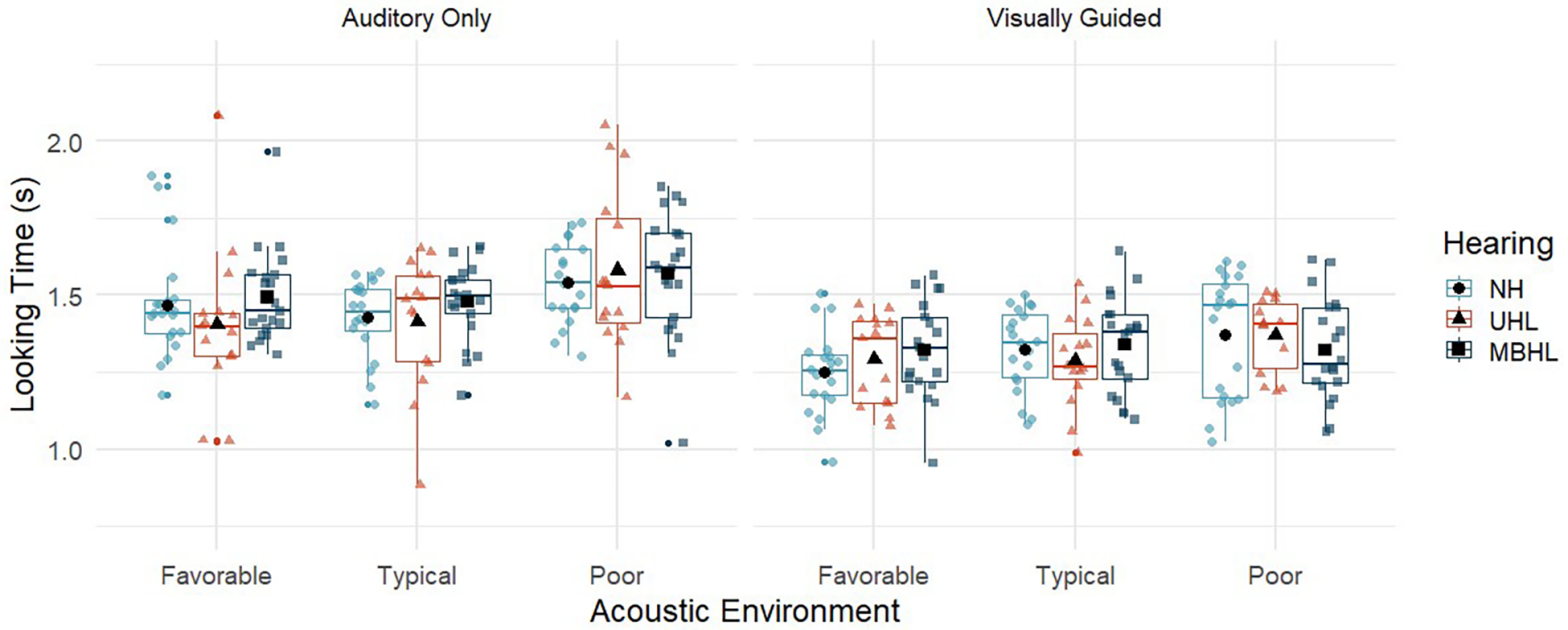
Looking time for children with normal hearing (NH; light blue circles), children with unilateral hearing loss (UHL; red triangles) and children with mild bilateral hearing loss (MBHL, dark blue squares) for the three acoustic environments (favorable, typical, poor). Results are shown for the Auditory-Only (left panel) and Visually Guided (right panel) conditions. Boxes represent the interquartile range, and whiskers represent the 5th and 95th percentiles. For each box, lines represent the median. Colored symbols represent individual data points. Black filled symbols represent means.

Figure 3 and Table 4 show looking time in seconds for each group across acoustic environments and auditory/visual cues. The pattern of looking time across group and conditions (Table 5) was the same as the percent correct looking results, with *post-hoc t*-tests showing that typical and favorable acoustic conditions were not different (Coefficient = .007, *p* = 0.66) but both had significantly shorter looking time than the poor condition (Coefficient = .08, *p* < 0.001). Visually guided conditions had shorter looking times than auditory-only conditions (Coefficient = −0.17, *p* < 0.001). There were no significant effects of hearing group, age, or higher-order interactions related to looking time in the localization-only task.

**Table 4:** Mean (standard deviation) for looking time (seconds) by group and listening condition.

Condition	NH	UHL	MBHL
Favorable (AO)	1.5 (0.2)	1.4 (0.3)	1.5 (0.2)
Favorable (VG)	1.3 (0.1)	1.3 (0.1)	1.3 (0.2)
Typical (AO)	1.4 (0.1)	1.4 (0.2)	1.5 (0.1)
Typical (VG)	1.3 (0.1)	1.3 (0.1)	1.3 (0.2)
Poor (AO)	1.5 (0.1)	1.6 (0.3)	1.6 (0.2)
Poor (VG)	1.4 (0.2)	1.4 (0.1)	1.3 (0.2)

AO, auditory only; VG, visually guided; NH, normal hearing, UHL, unilateral hearing loss; MBHL, mild bilateral hearing loss.

**Table 5:** Linear mixed effects model for group by condition.

Predictors	Looking time in seconds		
	Estimates	CI	*p*
(Intercept)	1.55	1.33–1.77	**<0**.**001**
Poor vs. favorable	0.08	0.05–0.12	**<0**.**001**
Typical vs. favorable	0.01	−0.03 to 0.04	0.657
AO vs. AV	−0.17	−0.20 to −0.14	**<0**.**001**
NH vs. MBHL	−0.02	−0.10 to 0.05	0.529
UHL vs. MBHL	−0.04	−0.12 to 0.04	0.377
Age (years)	−0.01	−0.03 to 0.01	0.479
Random effects			
σ^2^	0.02		
τ_00__ID_	0.01		
ICC	0.39		
N _ID_	56		
Observations	334		
Marginal R^2^/conditional R^2^	0.237/0.536		

AO, auditory only; VG, visually guided; NH, normal hearing, UHL, unilateral hearing loss; MBHL, mild bilateral hearing loss.

Estimates represent the coefficients for each variable in the model. For categorical predictors, the estimate represents the mean difference. For continuous predictors, the estimate represents the change in speech recognition for a one unit change in the predictor.

All *p*-values for significant effects are bolded.

*Do auditory and visual accessibility impact speech perception for children with MBHL, UHL, or NH and how does performance compare across groups?*

**Figure 4:**
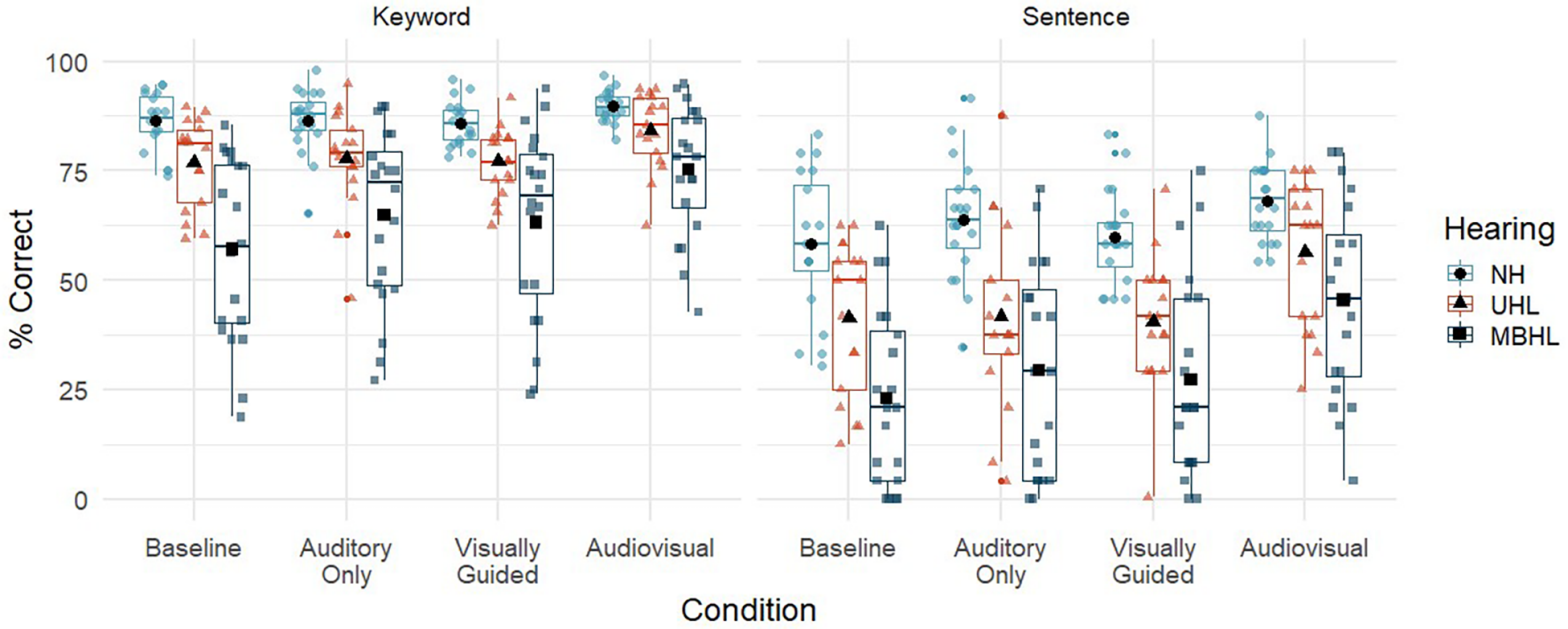
Speech perception (% correct) for children with normal hearing (NH; light blue circles), children with unilateral hearing loss (UHL; red triangles) and children with mild bilateral hearing loss (MBHL, dark blue squares) for the four listening conditions (auditory-only, visually guided, audiovisual, baseline). Results are shown for scoring by keyword (left panel) and sentence (right panel). Boxes represent the interquartile range, and whiskers represent the 5th and 95th percentiles. For each box, lines represent the median. Colored symbols represent individual data points. Black filled symbols represent means.

Figure 4 and Table 6 show speech perception in percent correct by scoring method (keyword vs. sentence) and conditions (auditory-only, visually guided, audiovisual, and single location baseline) for children with NH, UHL, or MBHL. The linear mixed effects models allow for a comparison of two different scoring methods (and their correlation within participants) on the outcome of the models. There could be differences in the model depending on whether the scoring was based on keywords correct or whether the entire sentence was correct. We included a term in the model to account for this potential effect. The main effect indicated that keyword scoring was approximately 30% better than the whole sentence scoring, but that none of the interactions depended on the scoring method. When accounting for this effect, we use the term speech recognition because the main effects of other variables reflect an overall composite of keyword and whole sentence scores for each participant. This can be interpreted that the main effects of group and condition were the same regardless of how the sentences were scored.

**Table 6:** Mean (standard deviation) for speech perception (%) by scoring method (keyword, sentence), group (NH, UHL, MBHL) and listening condition (baseline, AO, VG, AV).

Condition	NH	UHL	MBHL
Keywords			
Baseline	86.4 (6.5)	77.0 (10.1)	57.1 (20.8)
AO	86.5 (7.1)	77.8 (11.6)	64.8 (20.0)
VG	85.9 (4.9)	77.0 (7.8)	63.2 (21.7)
AV	89.8 (3.5)	84.3 (8.7)	75.3 (14.9)
Sentences			
Baseline	58.2 (16.1)	41.4 (17.6)	22.9 (20.3)
AO	63.9 (13.3)	41.7 (21.1)	29.4 (24.0)
VG	59.9 (10.6)	40.5 (15.4)	27.3 (23.0)
AV	68.1 (9.1)	56.4 (16.7)	45.4 (22.3)

AO, auditory only; VG, visually guided; AV, audiovisual; NH, normal hearing, UHL, unilateral hearing loss; MBHL, mild bilateral hearing loss.

Table 7 shows the statistics for the linear mixed effects model for speech recognition. The main effects of condition, hearing group, age, and scoring, and the condition by hearing group interaction were statistically significant. Percent correct looking was not associated with speech recognition. For every one-year increase in age, there was a 3.4% increase in speech recognition. *Post-hoc t*-tests were used to assess the effects of condition, hearing group and their interaction. Children with NH had speech recognition that was 27.1% higher (*p* < 0.001) than children with MBHL and 13.9% higher (*p* = 0.005) than children with UHL across conditions. Children with UHL had speech recognition that was 13.2% higher (*p* = 0.004) than children with MBHL across conditions. For each listening condition, the *post-hoc* tests were conducted in reference to the single location baseline condition, which was the condition with the poorest speech recognition across groups. Speech recognition in the auditory-only (+7.1%, *p* < 0.001) and visually guided (+5.3%, *p* < 0.001) conditions was significantly higher than the baseline condition. Speech recognition in the audiovisual condition was higher than the baseline (+20.3%, *p* < 0.001), auditory-only (+13.3, *p* < 0.001) and visually guided (+15%, *p* < 0.001) conditions. The significant interaction between hearing group and condition was driven by a larger difference in speech recognition between children with NH and children with MBHL in the audiovisual and baseline conditions than between children with NH and children with UHL in those conditions.

**Table 7:** Linear mixed effects models for group by condition.

Predictors	Sentences and keywords correct		
	Estimates	CI	*p*
(Intercept)	27.14	5.06–49.22	**0**.**016**
NH vs. MBHL	27.00	18.52–35.48	**<0**.**001**
UHL vs. MBHL	13.26	4.40–22.11	**0**.**003**
AV vs. AO	13.00	8.55–17.45	**<0**.**001**
Baseline vs. AO	−7.39	−11.98 to −2.80	**0**.**002**
VG vs. AO	−1.96	−6.09 to 2.17	0.351
Age (years)	3.40	1.36–5.43	**0**.**001**
Scoring	−30.65	−32.35 to −28.95	**<0**.**001**
Correct Looking	0.01	−0.07 to 0.09	0.784
NH AV vs. MBHL AV	−9.45	−15.19 to −3.71	**0**.**001**
UHL AV vs. MBHL AV	−2.72	−8.70 to 3.27	0.373
NH baseline vs. MBHL baseline	4.22	−1.52 to 9.97	0.149
UHL baseline vs. MBHL baseline	6.44	0.40–12.48	**0**.**037**
NH VG vs. MBHL VG	−0.45	−6.18 to 5.28	0.877
UHL VG vs. MBHL VG	0.81	−5.19 to 6.81	0.791
Random effects			
σ^2^	84.95		
τ_00__subid_	142.05		
ICC	0.63		
N_subid_	57		
Observations	456		
Marginal R^2^/conditional R^2^	0.649/0.868		

AO, auditory only; VG, visually guided; AV, audiovisual; NH, normal hearing, UHL, unilateral hearing loss; MBHL, mild bilateral hearing loss.

Estimates represent the coefficients for each variable in the model. For categorical predictors, the estimate represents the mean difference. For continuous predictors, the estimate represents the change in speech recognition for a one unit change in the predictor.

All *p*-values for significant effects are bolded.

*For children with MBHL or UHL, do audiological (audibility in better (MBHL) or poorer (UHL) ear) and cognitive (vocabulary, working memory) factors help to explain individual differences in speech perception?*

To examine the factors that led to individual differences in keyword recognition for children with UHL or MBHL, a separate linear mixed effects model was constructed with the same structure as the full model that included children with NH, but also included audiological variables, vocabulary, and working memory (Table 8). Degree of hearing loss was represented as the better-ear pure tone average for the children with MBHL and the poorer-ear pure tone average for the children with UHL. The main effects of this model mirrored the full model including children with NH. The degree of hearing loss was significantly related to keyword recognition, but there was a significant interaction with hearing group that suggested the pattern of degree of hearing loss and keyword recognition was different between children with MBHL and children with UHL. Specifically, the effect of degree of hearing loss on keyword recognition was stronger for children with MBHL than children with UHL. None of the other audiological factors, vocabulary, or working memory had a significant relationship with keyword recognition after controlling for other factors.

**Table 8:** Linear mixed effects model for children with MBHL or UHL.

Predictors	Keyword recognition for MBHL and UHL		
	Estimates	CI	*p*
(Intercept)	78.60	29.84–127.36	**0**.**002**
degree	−1.87	−2.57 to −1.17	**<0**.**001**
UHL vs. MBHL	−49.87	−76.02 to −23.72	**<0**.**001**
PPVT	0.18	−0.16 to 0.51	0.298
AWMAOdd	0.12	−0.13 to 0.37	0.331
Degree * hearing status [UHL]	1.86	1.14–2.58	**<0**.**001**
Random effects			
σ^2^	468.07		
τ_00__subid_	73.46		
ICC	0.14		
N_subid_	37		
Observations	296		
Marginal R^2^/conditional R^2^	0.244/0.346		

UHL, unilateral hearing loss; MBHL, mild bilateral hearing loss; PPVT, Peabody Picture Vocabulary Test; AWMAOdd, Odd One Out subtest for the Automated Working Memory Assessment. Estimates represent the coefficients for each variable in the model. For categorical predictors, the estimate represents the mean difference. For continuous predictors, the estimate represents the change in keyword recognition for a one unit change in the predictor.

All *p*-values for significant effects are bolded.

## Discussion

The current study examined the impact of MBHL or UHL on children's ability to locate and understand talkers under a range of acoustic and auditory/visual conditions. Identifying potential differences in performance across hearing status groups may help to guide intervention for these children.

The localization-only task addressed the ability of children with NH and children with MBHL or UHL to locate talkers who were presented auditory only or with a visual guide to the talker's location in three acoustic environments that children might experience in classrooms. Overall, children were better able to correctly locate talkers in the visually guided condition than in the auditory-only condition. This finding is consistent with findings from adults (38, 39), suggesting that visual information about a talker's location can improve localization of that talker for children with NH, MBHL, or UHL. There were no effects of age on looking behavior, suggesting that the children in the age range studied here were similarly adept at locating the talkers. The impact of acoustics on looking behavior were mixed. Overall, children correctly located talkers least in the poorest acoustic environment. The absence of a difference between typical vs. favorable acoustics suggests that children may be able to tolerate a range of acoustic environments without impacting their ability to find talkers in environments similar to the ones simulated in the current study. There also was no effect of hearing status. The absence of this effect was somewhat surprising, particularly for the poor acoustic condition where auditory access would be expected to have a greater impact on the two hearing-loss groups than on children with NH. However, the results suggest that even with reduced audibility, children with MBHL or UHL exhibited similar abilities to their peers with NH when attempting to locate talkers, suggesting that the task was not more difficult for them even with poorer auditory access. Although average percent correct looking scores were not significantly different across the three groups, the pattern of scores for the children with UHL in the auditory-only condition (see Figure 2), suggests a greater negative effect for some of these children when visual cues were unavailable. Studies using a greater variety of acoustic conditions and talker locations could be helpful in further differentiating potential hearing status effects on looking behaviors.

When children correctly located talkers, their looking times followed the same patterns as the correct looking scores. Looking times were shorter for visually guided than for auditory-only conditions and were longer in the poor auditory environment than in the typical and favorable environments. There were no effects of age or hearing group. Even with reduced auditory access, children with MBHL or UHL may not take longer to locate talkers than children with NH during some listening tasks.

Localization-only results suggest that children can benefit from the addition of visual information that guides them to talker locations across varying acoustic environments often found in educational settings, particularly in poor acoustics. Modifications as simple as having the teacher point to students who are raising their hands can give other children the opportunity to locate a particular talker before they speak. It also could be helpful to arrange desks in such a way that talkers are easily located (e.g., positioning in an arc rather than rows).

The speech perception task examined the ability of children with NH and children with MBHL or UHL to both locate multiple talkers and repeat back what those talkers said under varying auditory/visual conditions in a single acoustic environment. This task was not expected to be as difficult as the comprehension task used in our earlier study (12). However, it had the potential to address differences between children with MBHL and UHL that may have been masked by the difficulty of a complex comprehension task. It was anticipated that the syntactically correct/semantically incorrect sentences used in the current study would provide an additional level of difficulty over previous findings that used sentences that were both syntactically and semantically correct, and that differences in SNRs for children with NH vs. children with HL would avoid floor and ceiling effects for speech recognition.

Overall, the findings support other studies that have shown that speech understanding in noise for children with NH and children with hearing loss improves with age (51–54). Children's speech recognition was highest in the audiovisual condition and lowest in the baseline (single location at 0^o^ azimuth) condition. Speech recognition was better in the visually guided and auditory-only conditions than in the baseline condition but providing visual guidance to talker location did not improve speech recognition over auditory-only presentations. There was no significant effect of correct looking on speech recognition. These findings suggest that being able to find talkers more quickly does not necessarily result in better speech understanding if individuals do not see the talkers speaking once they have been located. It is possible that benefits of visual guidance for locating talkers will vary with the task. In tasks with high cognitive load, for example, visual guidance and audiovisual input could work together to improve speech understanding. Additional research would be needed to address this issue.

Poorer speech recognition in the baseline relative to the auditory-only condition was unexpected. In the baseline condition there was no need to locate talkers before repeating the sentences, potentially resulting in less listening effort than when talkers were in multiple locations. It is possible that children were less attentive in this condition, which they may have expected to be easier, than in conditions where they were required to find talkers. However, this could not be verified in the current study. Further research with this specific set of conditions and methodology is needed to address the issue.

As previously noted, the number of participants was motivated by a power analysis for main effects by group; however, we did not conduct a power analysis to determine how many participants would be required for group by condition interactions. Thus, it is possible that we may be underpowered for those comparisons. Many of the statistically significant effects observed in this study were small to medium effect sizes, suggesting there was sufficient power to address the research questions of interest.

Despite listening to speech at a poorer SNR, children with NH demonstrated better speech recognition than either children with MBHL or UHL. Seeing the talkers improved speech recognition for all groups, but children with MBHL or UHL continued to perform more poorly than their peers with NH, even in the audiovisual condition. These findings suggest that being able to see talkers as they are speaking is beneficial, but not sufficient to overcome reduced auditory access for children with MBHL or UHL. Children with UHL performed better than children with MBHL. In the current study, NH in one ear provided benefit for speech recognition in complex conditions that was not available for children with mild hearing loss in both ears.

Factors that may impact individual differences in speech understanding were examined for the children with MBHL or UHL. Only degree of hearing loss was shown to have a significant effect. Degree of hearing loss in the better ear of children with MBHL had a greater impact on keyword understanding than did degree of hearing loss in the poorer ear for children with UHL. This occurred despite a better mean and smaller range of BEPTAs for the children with MBHL than for PEPTAs in the children with UHL (see Table 1). These findings support the benefit of NH on speech perception, even when that NH occurs in only one ear.

Previous research has suggested that degree of hearing loss in the poorer hearing ear may impact speech perception and localization abilities in children with UHL (23). Although population-based studies show poorer ear thresholds in children with UHL are equally represented across a wide range of severity levels (55, 56), the hearing loss levels of participants in individual studies, including the current study, may not include similar numbers of children representing this wide range of severity levels (57). Further research that includes a larger number of children across a representative range of severity for the poorer ear is needed to further address how degree of hearing loss in the poorer ear may differentially impact outcomes in children with UHL.

Hearing aids may improve auditory access for children with MBHL or UHL (58–60); however, there is currently no clear consensus regarding personal amplification recommendations for these populations and both hearing aid recommendations and hearing aid use may be delayed and/or inconsistent (55, 61–64). In the current study, all children were tested without amplification to represent potential worst-case outcomes based on hearing status. Future studies in complex conditions reflecting real-world listening should include measures with amplification to address how improving audibility, in both ears for children with MBHL or one ear for children with UHL who are able to use a hearing aid in the poorer hearing ear, can impact outcomes. Such studies should also examine consistency of hearing aid use in children who are fitted with personal amplification to determine potential effects on outcomes.

## Data Availability

The data presented in this article are not readily available as per ethics approvals. Requests to access the data should be directed toward the corresponding author.
